# Genome Survey Sequencing for the Characterization of the Genetic Background of *Rosa roxburghii* Tratt and Leaf Ascorbate Metabolism Genes

**DOI:** 10.1371/journal.pone.0147530

**Published:** 2016-02-05

**Authors:** Min Lu, Huaming An, Liangliang Li

**Affiliations:** 1 Agricultural College, Guizhou University, Guiyang, Guizhou, China; 2 Guizhou Engineering Research Center for Fruit Crops, Guiyang, Guizhou, China; Jawaharlal Nehru University, INDIA

## Abstract

*Rosa roxburghii* Tratt is an important commercial horticultural crop in China that is recognized for its nutritional and medicinal values. In spite of the economic significance, genomic information on this rose species is currently unavailable. In the present research, a genome survey of *R*. *roxburghii* was carried out using next-generation sequencing (NGS) technologies. Total 30.29 Gb sequence data was obtained by HiSeq 2500 sequencing and an estimated genome size of *R*. *roxburghii* was 480.97 Mb, in which the guanine plus cytosine (GC) content was calculated to be 38.63%. All of these reads were technically assembled and a total of 627,554 contigs with a N50 length of 1.484 kb and furthermore 335,902 scaffolds with a total length of 409.36 Mb were obtained. Transposable elements (TE) sequence of 90.84 Mb which comprised 29.20% of the genome, and 167,859 simple sequence repeats (SSRs) were identified from the scaffolds. Among these, the mono-(66.30%), di-(25.67%), and tri-(6.64%) nucleotide repeats contributed to nearly 99% of the SSRs, and sequence motifs AG/CT (28.81%) and GAA/TTC (14.76%) were the most abundant among the dinucleotide and trinucleotide repeat motifs, respectively. Genome analysis predicted a total of 22,721 genes which have an average length of 2311.52 bp, an average exon length of 228.15 bp, and average intron length of 401.18 bp. Eleven genes putatively involved in ascorbate metabolism were identified and its expression in *R*. *roxburghii* leaves was validated by quantitative real-time PCR (qRT-PCR). This is the first report of genome-wide characterization of this rose species.

## Introduction

Presently, about 100–250 species have been described in the genus *Rosa*, many of which are recognized for their ornamental horticultural use [1]. The chromosome number of members of this genus are based on multiples of seven and range from 2n = 2x = 14 to 2n = 8x = 56 [2]. *Rosa roxburghii* Tratt (2n = 2x = 14), which is widely distributed in Southwest China, has aroused statewide interest for its wide range of nutritional and medicinal components in fruits as well as in leaves, including ascorbate (AsA), superoxide dismutase, flavonoids, and polysaccharides [3–5]. The economic cultivation area of this species in China involves at least 30,000 hectares, and a series of health care products has been developed for clinical applications.

Despite its economic importance, the inheritance pattern of most agronomically significant traits of *Rosa roxburghii* has not yet been established. The limited genetic and genomic resources for this species have thus resulted in minimal improvement in its breeding programs. Collecting wild germplasm and selecting elite genotypes of this rosebush based on plant growth vigor and fruit characteristics started in the early 1980s in China [6], and only one cultivar and some elite lines have been identified to date [7]. Random amplification of polymorphic DNA (RAPD) and amplified fragment length polymorphism (AFLP) [8] markers have been employed to describe the genotypes of *Rosa roxburghii*. Recently, several SSR markers have been developed based on transcriptome sequencing [9], but no genomic sequence-based markers are available for this species.

**Fig 1:**
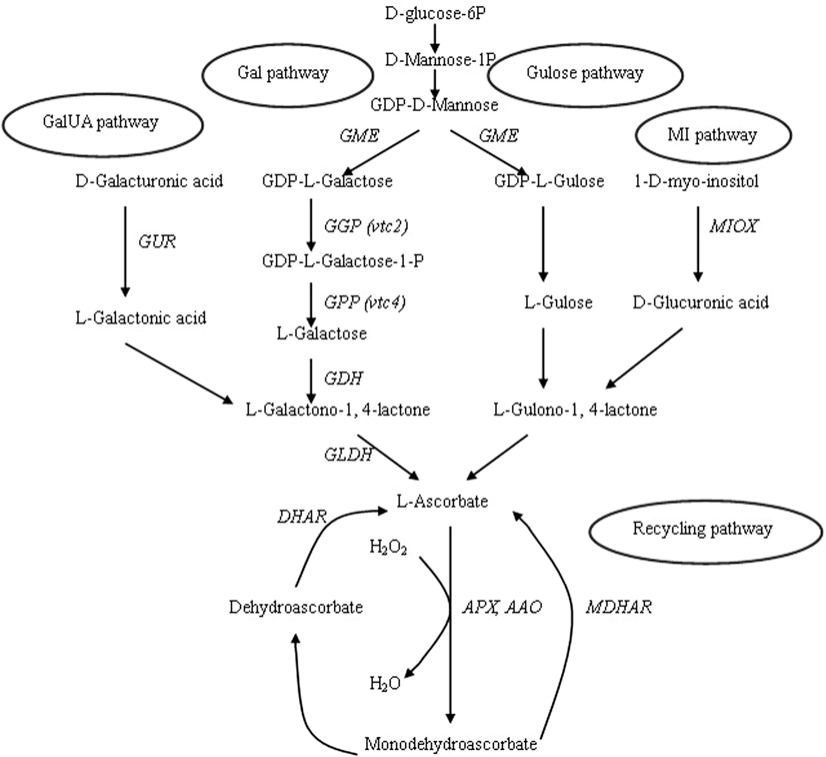
The proposed AsA synthetic and recycling pathways in higher plants. The four pathway included GalUA (D-galacturonic acid) pathway, Gal (L-galactose) pathway, Gulose(L-gulose) pathway and MI (Myo-inositol) pathway which catalyzed by GUR (D-galacturonate reductase), GME (GDP–D–Mannose-3,5-epimerase), GGP (GDP-L-galactose guanyltransferase), GPP (L-galactose-1-phosphate phosphatase), GDH (L-galactone dehydrogenase), GLDH (L-Galactono-lactone dehydrogenase) and MIOX(myo-inositol oxygenase). The recycling pathways were catalyzed by APX (ascorbate peroxidase), AAO (ascorbate oxidase), MDAR (mono-dehydroascorbate reductase) and DHAR (dehydroascorbate reductase).

AsA, also known as vitamin C, is of vital importance to plant cells as an antioxidant and enzyme cofactor [10, 11]. Several AsA biosynthetic pathways have been proposed in higher plants and the route that occurs via L-galactose has been well established [12]. Recently, we identified and analyzed the candidate genes involved in the biosynthesis of AsA in the *R*. *roxburghii* fruit based on the fruit transcriptome data [9]; however, the metabolic mechanisms underlying AsA overproduction in this plant remain unknown. In addition, the level and distribution of AsA generally depends on both its synthesis as well as recycling [13]. Biosynthesized AsA can be oxidized to mono-dehydroascorbate (MDA) and ultimately to dehydroascorbate (DHA) by the activities of ascorbate peroxidase (APX; EC 1.11.1.11) and ascorbate oxidase (AAO; EC 1.10.3.3). Then, part of the oxidized AsA is reduced back to AsA through the ascorbate–glutathione cycle by MDA reductase (MDAR; EC 1.6.5.4) and DHA reductase (DHAR; EC 1.8.5.1) [11]. The proposed AsA synthetic and recycling pathways were shown in the Fig 1.

Following the success of the Human Genome Project, several Rosaceae species, including *Malus* × *domestica* [14], *Fragaria vesca* [15], *Prunus mume* [16], *Prunus persica* [17], *Pyrus bretschneideri* [18], and *Pyrus communis* [19] have been sequenced by using next-generation sequencing (NGS) technology. Genome survey sequencing via NGS is an important and cost-effective strategy in generating extensive genetic and genomic information relating to the metabolism and development of organisms. Therefore, to investigate and provide a genomic resource of this species, we conducted a genome survey of *R*. *roxburghii* using NGS. Based on these data, we identified candidate genes involved in leaf AsA metabolism. The results of the present study contribute to accelerating the progress of gene discovery, genetic diversity, evolutionary analysis, structural genomic studies, and genetic improvement of *R*. *roxburghii*, as well as its closely related species.

## Materials and Methods

### Plant materials

Plants of *R*. *roxburghii* ‘Guinong 5’ [7] were grown in the fruit germplasm repository of Guizhou University, Guiyang, China (26°42.408'N, 106°67.353'E). Genomic DNAs were isolated from young leaf tissues of *R*. *roxburghii* using a plant genomic DNA extraction kit (Tiangenbiotech, Beijing, China), following the manufacturer’s instructions. DNA quality and quantity were assessed by 1% agarose gel electrophoresis, and the concentrations of nucleic acids and proteins were measured on a BioPhotometer (Eppendorf, Germany).

### Genome sequencing and genome size estimation

Paired-end library with insert size of 220 base pairs (bp) was constructed from randomly fragmented genomic DNA, following the standard protocol (Illumina, Beijing, China). Sequence data was generated by Beijing Biomarker Technologies Co., Ltd. (Beijing, China), using an Illumina HiSeq 2500 sequencing platform. The read length was 126 bp, and clean reads were obtained after filtering and correction of the sequence data, and were relatively accurate for estimating the size of the genome, repetitive sequences, and heterozygosis. Then, based on K-mer analysis, information on peak depth and the number of 17-mers was obtained. Its relationship was expressed by using the following algorithm: Genome size = K-mer num/Peak depth [20].

### Sequence assembly and guanine plus cytosine (GC) content analysis

SOAPdenovo software [21] and Abyss were applied for genome assembly with the pre-processed reads, where k-mer sizes of 31, 54, 63, 70, 77, and 83 were examined using default parameters, and the optimal k-mer size was selected from the N50 length. The usable reads > 200 bases in length were selected to realign the contig sequences because the sequences < 200 bp were likely to be derived from repetitive or low-quality sequences. Then, the paired-end relationship between reads was coincident between contigs. The scaffolds were constructed step by step using insert size paired-ends. The 10-kb non-overlapping sliding windows along the assembled sequence were used to calculate GC average sequencing depth.

### Repetitive sequences

Due to the relatively low conservatism of the repetitive sequence among species, a particular repetitive sequence database was built to predict repeat sequences. The software programs LTR_FINDER [22], MITE-Hunter [23], RepeatScout [24], and PILER-DF [25] were used to construct a *de novo* repeat library, classified by PASTEClassifier [26], and combined with the Repbase transposable element library [27] to act as the final library. Then, the software RepeatMasker [28] was run to find homologous repeats in the final library. SSR motifs were identified using the SciRoKo software [29] in the ‘MISA’ mode, with default parameters. The minimum numbers of SSR repeats for mono-, di-, tri-, tetra-, penta-, and hexa-nucleotides adopted for identification were 14, 7, 5, 4, 4, and 4, respectively.

### Gene prediction and annotation

For *de novo* prediction, after filtering scaffolds of < 1000 bp in size, Genscan [30] and Augustus were used to predict genes with parameters trained on *R*. *roxburghii*. Then, BLAST alignment was performed between predicted genes and common databases such as Nt, Nr, TrEMBL, Swiss-Prot, Pfam, ‘euKaryotic clusters of Orthologous Groups’ (KOG), Kyoto Encyclopedia of Genes and Genomes (KEGG), plant Gene ontology (GO), and Clusters of Orthologous Groups (COG). Meanwhile, the described genes were classified into the KOG slim categories, the GO categories, and then mapped onto the KEGG reference pathways as described by Hirakawa *et al*. [31]. For homology-based prediction, protein sequences for *Malus×domestica*, *Pyrus bretschneideri*, *Fragaria vesca*, *Prunus mume*, *Prunus persica*, and *Vitis vinifera* were downloaded from publicly available databases. The putative genes of *R*. *roxburghii* were clustered by using OrthoMCL [32] with the unigene sets of apple, pear, strawberry, *Prunus mume*, and peach. Single-copy protein sequences of *R*. *roxburghii* and the 6 other species were used to construct the evolutionary tree by using the software PHYML [33].

### Genes involved in AsA metabolism

AsA and DHA were measured according to the method described by An *et al*. [34]. For qRT-PCR validation, 11 cDNAs encoding GDP-mannose-3',5'-epimerase (GME), GDP- L-galactose-1-phosphate phosphorylase (GGP), L-galactose-1-phosphate phosphatase (GPP), L-galactose dehydrogenase (GDH), L-galactono-1,4-lactone dehydrogenase (GLDH), D-galacturonate reductase (GUR), *myo*-inositol oxygenase (MIOX), AAO, APX, DHAR, and MDHAR proteins, all of which have potential roles in AsA metabolism, were selected. Target gene primers were designed (S1 Table) according to acquired sequences using the Primer Express software (Applied Biosystems, USA). Total RNAs were extracted from *R*. *roxburghii* leaves at a leaf age of 10 days while fully expanding, 50-day-old leaves were labeled as mature, and 90-day-old leaves were designated as aged, using the TRIzol reagent (Invitrogen), followed by purification with an RNA purification kit (Takara). qRT-PCR and subsequent data analysis was performed according to the method described by Yan *et al*. [9].

## Results

### Genome sequencing and genome size estimation

**Fig 2:**
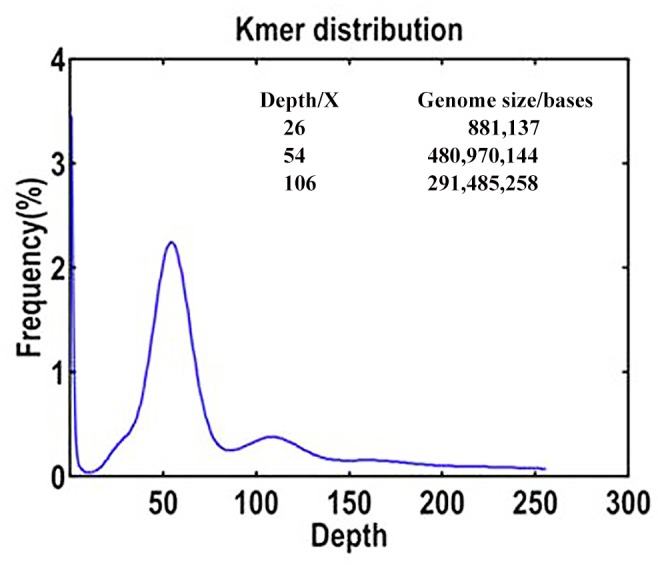
K-mer (k = 17) analysis for estimating the genome size of *R*. *roxburghii*. The x-axis is depth (X); the y-axis is the proportion that represents the frequency at that depth divided by the total frequency of all depths. The genome size was estimated by using the formula: Genome size = K-mer num/Peak depth, and the heterozygosis rate causes a sub-peak at a position half of that of the main peak, whereas a certain repeat rate can cause a similar peak at the position of multiple integers of the main peak.

After the sequence data was filtering and correction, a total of 30.29 Gb clean reads were generated from the small-insert (220 bp) library, with 95.14% Q20 bases (base quality > 20), about 62.99× coverage (Table 1), much greater than 30× coverage, which was required for successful assembly. All of the clean data were used for K-mer analysis. For the 17-mer frequency distribution (Fig 2), the number of K-mers was 26,445,309,972, and the peak of the depth distribution was at 54.98×. The estimated genome size was 480.97 Mb, which was calculated by using the following formula: Genome size = K-mer num/Peak depth. Similarly, a certain repeat rate could cause a repeat peak at the position of the integer multiples of the main peak, ~106×, so the genome size of repetitive sequences was estimated to be 291.49Mb, which was about 60.60% of the *R*. *roxburghii* genome. In addition, the heterozygosis rate could cause a sub-peak at a position half of the height of the main peak, ~26×, which indicates about 0.18% of the heterozygosis rate in this genome.

**Table 1:** Statistics of sequencing data.

Library	Read Length/bp	Data/bp	Depth/X	Q20 (%)	Q30 (%)
220 bp	126	30,294,326,779	62.99	95.14	91.25

### Sequence assembly and GC content analysis

All of the clean reads and the software SOAPdenovo and Abyss were used to carry out *de novo* assembly. Assembly with k-mer 77 by SOAPdenovo was selected, as it has the optimal reading for N50 (S2 Table), which is defined as a weighted median and is the smallest contig/scaffold size in the set whose combined length totals 50% of the genome assembly, to produce a contig with the N50 of ~1.48 kb, and a total length of ~ 405.81 Mb (Table 2). A sequence was also generated, with the scaffold N50 length of ~3.55kb and a total length of ~409.36 Mb. The total gap length (Ns) was ~3.55 Mb.

**Table 2:** Statistics of the assembled genome sequences.

**Contigs**	
Number of sequences	627,554
Total length (bases)	405,809,290
N50 length (bases)	1,484
N90 length (bases)	236
Number of sequences ≥500 bp	183,973
Number of sequences ≥1 kb	94,798
Number of sequences ≥10 kb	1,224
Number of contigs in scaffolds	415,383
Number of contigs not in scaffolds	963,281
**Scaffolds**	
Number of sequences	335,902
Total length (bases)	409,356,560
N50 length (bases)	3,554
N90 length (bases)	375
Number of sequences ≥500 bp	143,058
Number of sequences ≥1 kb	84,286
Number of sequences≥10 kb	5,071
A	125,078,917
T	123,942,623
G	77,958,241
C	78,829,509
N	3,547,270
Total (ACGT)	405,809,290
G+C% (ACGT)	38.64

The N50 of contigs and scaffolds was calculated by ordering all sequences, then adding the lengths from the longest to shortest until the added length exceeded 50% of the total length of all sequences. N90 is similarly defined.

**Fig 3:**
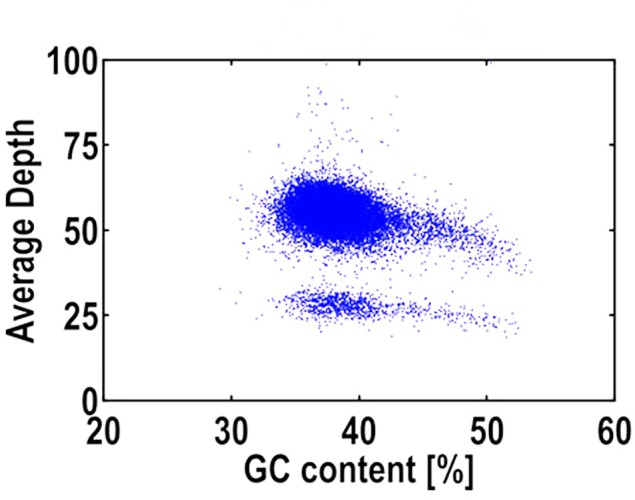
GC content and average sequencing depth of the genome data used for assembly. The x-axis was GC content percent across every 10-kb non-overlapping sliding window.

The average GC content of *R*. *roxburghii* genome was ~ 38.64% (Table 2), which was higher than that of ants (33.7–37.7%) [35, 36] and potatoes (34.8–36.0%) [31, 37], lower than that of human (41%) and *Nasonia vitripennis* (40.6%) [38], but similar to that of date palm (38.5%) [39] and Australian kangaroo (38.8%) [40]. Therefore, the *R*. *roxburghii* genome was of mid-GC content. A too high (>65%) and too low (<25%) GC content may cause sequence bias on the Illumina sequencing platform, thus seriously affecting genome assembly [41]. Moreover, the GC depth was slightly blocked into 2 layers (Fig 3), which was in part caused by a 0.18% heterozygosity rate. Maybe only one of the two sets of homologous chromosomes in the diploid was assembled, which resulted in the emergence of the lower layer [21].

### Repetitive sequences

The total length of repetitive sequences was ~147.89 Mb (Table 3), which was about 47.55% of the *R*. *roxburghii* genome, and lower than that of other plant species such as pear (51.3%) [18], *Lotus japonicus* (56.8%) [42], potato (64.2%) [37], apple (67%) [14], tomato (68.3%) [43]. In addition, this length was also lower than the estimated number of K-mer (60.60%; Fig 2), which could be the limitations of the assembling effect that resulted in the loss of 21.54% of the repetitive sequences during assemble.

**Table 3:** Statistics of repetitive sequence.

Type		Number	Length (bp)	Rate (%)
Class I	DIRS	5,755	4,175,268	1.34
	LINEs	17,825	6,689,518	2.15
	LTRs	582	252,704	0.08
	LTRs/Copia	55,400	30,357,977	9.76
	LTRs/Gypsy	39,913	21,467,074	6.90
	PLE|LARD	25,914	7,763,942	2.50
	SINEs	25,918	5,001,722	1.61
	SINEs|TRIMs	100	27,816	0.01
	TRIMs	2,982	1,161,230	0.37
	Unknown	1,388	552,597	0.18
Class II	Cryptons	13	778	0.00
	Helitrons	5,893	1,966,508	0.63
	MITEs	23,940	5,015,314	1.61
	Mavericks	47	13,970	0.00
	TIRs	1,890	461,658	0.15
	TIRs/CACTAs	2,869	585,595	0.19
	TIRs/Ps	34	2,053	0.00
	TIRs/PIF-Harbinger	4,079	1,043,267	0.34
	TIRs/PiggyBac	16	798	0.00
	TIRs/Tc1-Mariner	168	21,090	0.01
	TIRs/hAT	8,652	2,015,525	0.65
	Unknown	12,947	2,266,751	0.73
	Potential Host Gene	3,067	827,410	0.27
	SSRs	48,270	3,587,743	1.15
	Unknown	234,148	52,634,201	16.92
Total		521,810	147,892,509	47.55

90.84 Mb transposable elements (TE) were obtained, comprised 29.20% of the genome (Table 3), in which retroelements and DNA transoson were identified. Retroelements, also called class I transposable element (Table 3), comprised 24.90% of the genome. And DNA transposons, also named class II transposable element (Table 3), comprised only 4.30% of the genome. Long terminal repeats (LTRs) were observed to be the most abundant repeat elements, comprised 16.74% of the genome, in which 6.90% was *gypsy*, 9.76% was *copia* and other LTRs occupied only 0.08% (Table 3). The ratio (0.71:1) of *gypsy*-like to *copia*-like elements was calculated. There were 1.15% SSRs and 16.92% uncharacterized repeats (Table 3).

**Fig 4:**
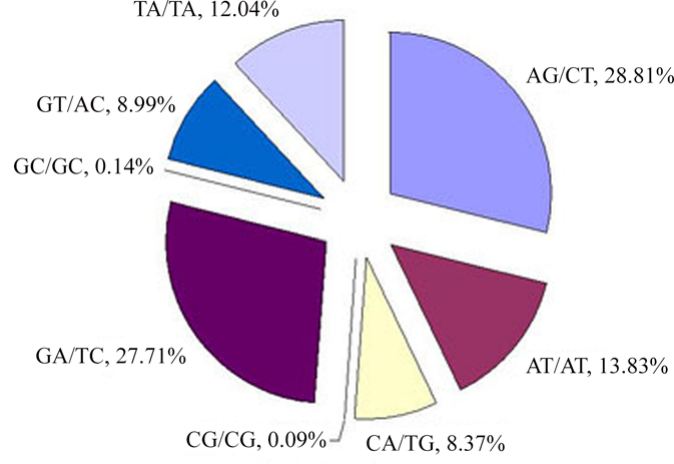
Percentage of different mofits in dinucleotide repeats in *R*. *roxburghii*.

**Fig 5:**
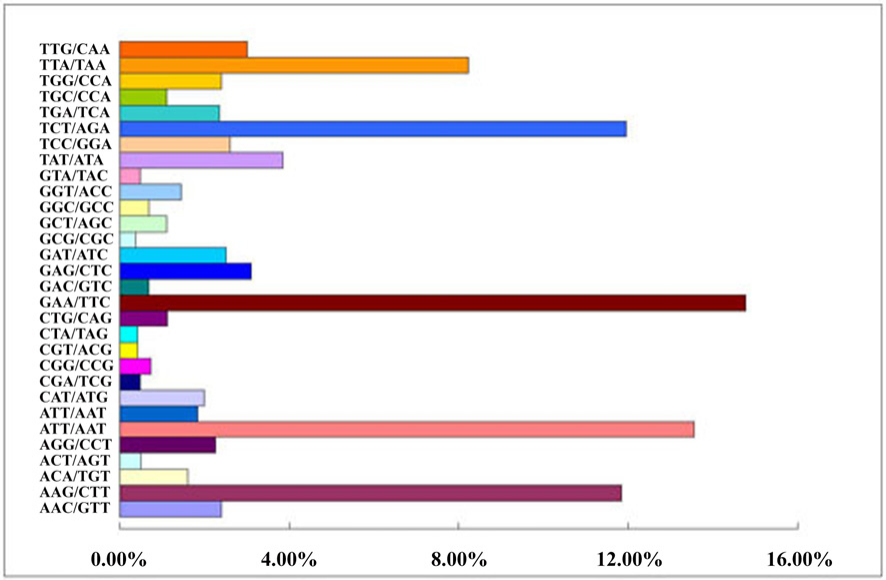
Percentage of different motifs in trinucleotide repeats in *R*. *roxburghii*.

A total of 167,859 SSRs were identified and among which mono-nucleotide repeats showed predominant type, which accounted for 66.30% of the observed SSRs, followed by the di- (25.67%), tri- (6.64%), tetra- (1.08%), penta- (0.16%), and hexa- (0.15%) nucleotide repeats (Table 4). Mono-nucleotide repeats have been reported to be the most common type of repeats whether in monocot species, such as rice, sorghum, and *Brachypodium* or in dicot species, for example, *Arabidopsis*, *Medicago*, and *Populus*, which accounted for 79% in *Medicago* at most [44]. The mono-, di- and tri-nucleotide repeats contributed to nearly 99% of SSRs in *R*. *roxburghii*, and a very small portion was contributed by tetra-, penta- and hexa-nucleotide repeats. Moreover, 363 motif types were identified in *R*. *roxburghii* genome, including 2 of mono-, 8 of di-, 30 of tri-, 80 of tetra-, 91 of penta-, and 152 of hexa-nucleotide repeats (S3 Table). Within the dinucleotide repeat motifs, the AG/CT was most abundant, which accounted for 28.81%, followed by GA/TC at 27.71% (Fig 4). And among the trinucleotide repeat motifs, the common motifs were GAA/TTC and ATT/AAT, accounting for 14.76% and 13.55%, respectively (Fig 5).

**Table 4:** Simple sequence repeat types detected in the *R*. *roxburghii* sequences.

Searching Item	Number	Ratio
Total number of sequences examined	84,355	
Total size of examined sequences (bp)	311,013,596	
Total number of identified SSRs	167,859	100.00%
Number of SSR containing sequences	56,364	33.58%
Number of sequences containing more than 1 SSR	36,597	21.80%
Number of SSRs present in compound formation	20,558	12.25%
Mono nucleotide	111,292	66.30%
Di nucleotide	43,083	25.67%
Tri nucleotide	11,149	6.64%
Tetra nucleotide	1,811	1.08%
Penta nucleotide	269	0.16%
Hexa nucleotide	255	0.15%

### Gene prediction and annotation

Based on the genome of *R*. *roxburghii*, with a filtering scaffold of < 1,000 bp for *de novo* prediction, program Augustus got a predicted gene number of 20,589, and a total of 22,721 genes were predicted by Genescan (Table 5). We choose Genescan for further analyses. The identified genes have an average length of 2,311.52 bp, an average exon length of 228.15 bp, and intron length of 401.18 bp. The number of predicted genes in the genome of *R*. *roxburghii* was much lower than that of other sequenced genomes such as *Malus×domestica* (57,386) [14], *Pyrus bretschneideri* (42,812) [18], *Fragaria vesca* (34,809) [15], *Prunus mume* (31,390) [16], and *Prunus persica* (27,852) [17]. It has been reported that the insufficient sequence depth coverage, variable regulation of gene expression levels, and low sequence homology because of limited gene information from closely related species might be possible reasons [45].

**Table 5:** Statistics of gene information.

Software	Gene number	Gene	Average gene	Exon	Average exon	Intron	Average Intron
		length (bp)					
Genscan	22,721	52,520,032	2311.52	19,040,306	228.15	33,479,726	401.18

**Fig 6:**
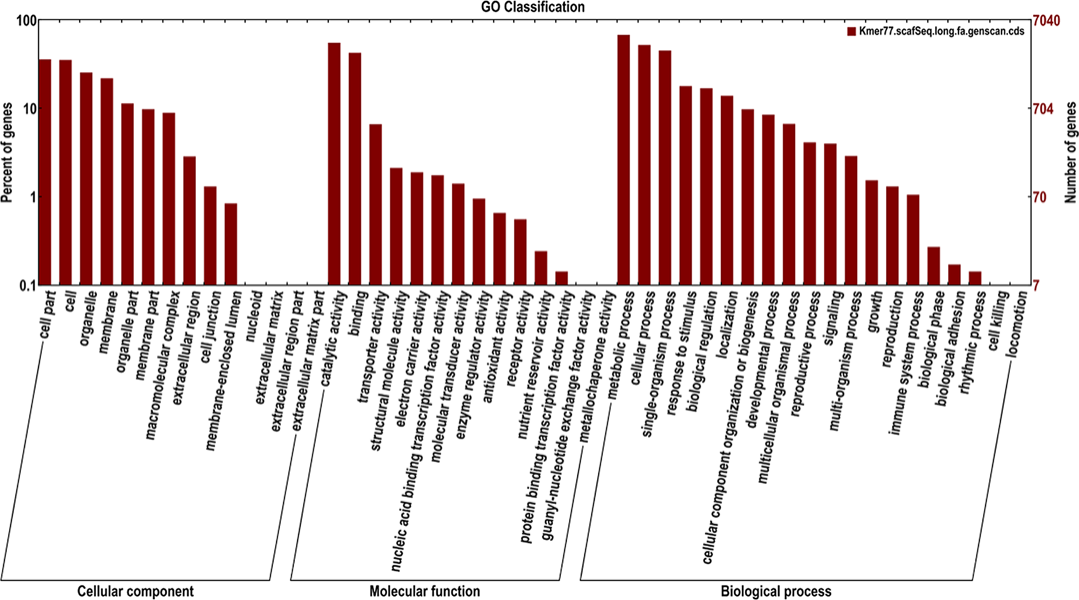
Gene Ontology classification. Genes were assigned to three categories: cellular components, molecular functions, and biological process.

Of the 22,721 predicted genes in the *R*. *roxburghii* genome, 17,637 genes matched known genes in common databases, of which, 11,622 had Swiss-Prot homologs, 16,173 had TrEMBL homologs, and 23.38% (5084) were unknown (Table 6). A total of 7,040 genes were identified by GO slim analysis and further classified into the categories of molecular function, cellular component, and biological process (Fig 6). First of all, around 48.70% of the genes were grouped under biological processes, in which metabolic process was the most highly represented group. Secondly, 29.46% of the genes were grouped under cellular components, in which cell part and cell were the most significantly represented groups. Finally, 21.84% of the genes were grouped under molecular functions, in which catalytic activity represented a relatively high proportion.

**Table 6:** Statistics of gene functional annotation.

Annotation database	Annotated Number	Percentage
COG	4,803	21.14%
GO	7,040	30.98%
KEGG	3,130	13.78%
KOG	8,404	36.99%
Pfam	11,414	50.24%
Swiss-Prot	11,622	51.15%
TrEMBL	16,173	71.18%
Nr	16,690	73.46%
Nt	15,909	70.02%
All	17,637	77.62%

A total of 8,404 putative genes were classified into KOG functional categories, the cluster for general function prediction only represented the largest group (1,986; 23.63%), followed by signal transduction mechanisms (994; 11.83%) and posttranslational modification, protein turnover, chaperones (941; 11.20%) (S1 Fig).

There were 3,130 putative genes assigned to 116 KEGG pathways (S4 Table). A total of 1,828 genes (58.40%) were associated with 84 metabolic pathways, in which 430 (23.52%) were involved in carbohydrate metabolism, followed by amino acid metabolism (321; 17.56%), energy metabolism (182; 9.96%), nucleotide metabolism (131; 7.17%), glycan biosynthesis and metabolism (122; 6.68%), biosynthesis of other secondary metabolites (121; 6.62%), lipid metabolism (118; 6.46%), metabolism of cofactors and vitamins (112; 6.13%), metabolism of other amino acids (108; 5.91%), glycan biosynthesis and metabolism (100; 5.47%), and metabolism of terpenoids and polyketides (83; 4.54%). In addition, 941 genes were associated with genetic information processing, 147 with environmental information processing, 135 with cellular processes, and 124 with organismal systems.

**Fig 7:**
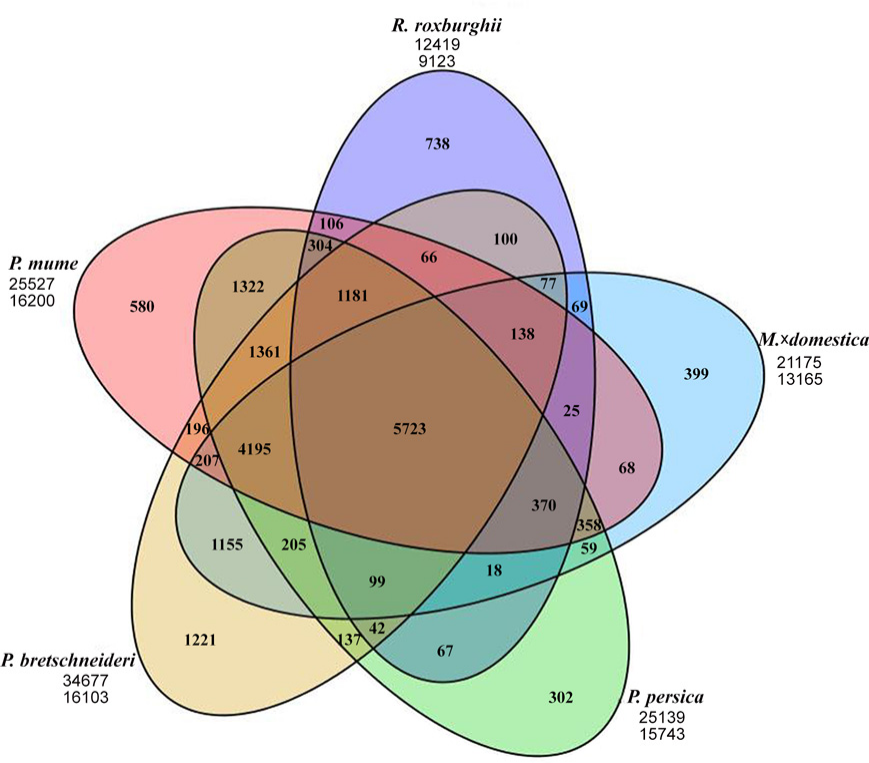
Venn diagram showing the number of gene clusters in *R*. *roxburghii* and other close species, i.e., *M*.*×domestica*, *P*. *persica*, *P*. *bretschneideri*, and *P*. *mume*. The first number under the species name is the total number of putative genes subjected to clustering. The second number is the clustered family number. The overlapping areas represent sequences clustered with other species, and the number of non-overlapping areas represents specific genes.

Of the putative *R*. *roxburghii* genes, 12,419 were clustered with predicted genes that were identified in other species, whereas the remaining 738 were not clustered and therefore considered as *R*. *roxburghii*-specific genes (Fig 7), which was far more than that of *Prunus persica* (302), *Malus×domestica* (399), and *Prunus mume* (580), but much lower than that of *Pyrus bretschneideri* (1,221). The evolutionary relationships among species (S2 Fig) proved that there was a closer relationship between rosebush *R*. *roxburghii* and herbaceous strawberry.

### Putative genes associated with AsA metabolism

Based on the genome survey sequencing dataset, 17 unique sequences were annotated as paralogs of 11 genes associated with AsA metabolism. Of these 17 sequences, two (Roxburghii008246-TA and Roxburghii018888-TA), three (Roxburghii002764-TA, Roxburghii-016536-TA, and Roxburghii016678-TA), and four (Roxburghii008076-TA, Roxburghii015197-TA, Roxburghii016587-TA, and Roxburghii018922-TA) were annotated as paralogs of *MDHAR*, *MIOX*, and *APX*, respectively, and the other 8 were in one-to-one correspondence (S5 Table).

To confirm experimentally that the genes obtained from sequencing were actually expressed, all of the 11 putative genes involved in AsA biosynthesis, namely, *GME* (Roxburghii013562-TA), *GGP* (Roxburghii021760-TA), *GPP* (Roxburghii007418-TA), *GDH* (Roxburghii012479-TA), *GLDH* (Roxburghii012337-TA), *MIOX* (Roxburghii002764-TA), *GUR* (Roxburghii012921-TA); in AsA oxidation, namely, *AAO* (Roxburghii002218-TA) and *APX* (Roxburghii008076-TA); and AsA recycling, including *DHAR* (Roxburghii013431-TA) and *MDHAR* (Roxburghii-008246-TA), were analyzed by qRT-PCR across three leaf developmental ages.

**Fig 8:**
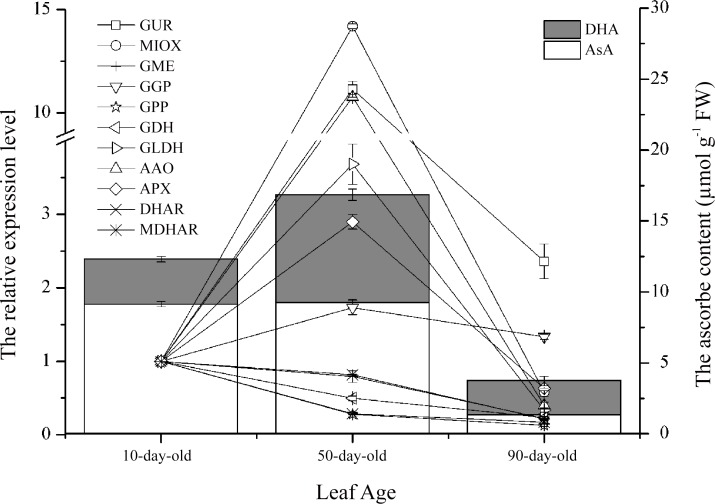
Relative expression of genes related to ascorbic acid metabolism during *R*. *roxburghii* leaf development. The *UBQ* gene was used as internal control, and the levels of expression of the target gene in fully expanding leaf samples were normalized to 1.0. The data for each sample are represented by the means of three replicates.

Fig 8 shows that all selected genes were expressed at varying levels during the three developmental stages, in which the expression of three genes involved in AsA synthesis, namely, *GLDH*, *GUR*, and *MIOX*, and two genes involved in AsA degradation, *AAO* and *APX*, reached highest abundance in mature leaves, and then markedly decreased until these aged. Similarly, leaf DHA and T-AsA (AsA + DHA) levels increased with leaf development, reaching its peak levels in mature leaves and then rapidly decreased. These results suggest that the AsA pool size in *Rosa roxburghii* leaves were regulated by biosynthesis, as well as recycling.

## Discussion

Flow cytometry has been regarded as a standard method for the prediction of the genome size of plants [46]. However, in the recent years, the development of the NGS technology has provided researchers an affordable means of addressing a wide range of questions relating to emerging and non-model species. In addition, the k-mer method has been successfully applied for the estimation of genome size using NGS reads without prior knowledge of the genome size. Such approach has been utilized in the analysis of the genomes of *Gracilariopsis lemaneiformis* [45], *Cucumis sativus* [47], and *Myrica rubra* [48]. The genome size estimated by K-mer depth distribution of sequenced reads is generally consistent with that of flow cytometry [14, 47]. In the present study, the estimated genome size of *R*. *roxburghii* was 480.97 Mb, which was close to the results estimated by flow cytometry (464.55 Mb) [49, 50].

Fruit trees are perennial, and the majority of these are highly heterozygous; therefore, the assembly of fruit tree genomes is relatively difficult using the WGS strategy. Homozygous materials for genome sequencing were always in priority [17, 45, 51], although a complicated bacterial artificial chromosome (BAC) approach could resolve problems associated with the assembly of a heterozygous genome [18]. Based on the feasibility of estimating heterozygosity from low-coverage genome sequence [52], a heterozygosity rate of 0.18% was observed in the *R*. *roxburghii*, which was higher than that of other sequenced plants such as pigeon pea (0.067%) [53], *Prunus mume* (0.08%) [16], but much lower than that of black cottonwood (0.26%) [54] and date palm (0.46%) [39], which could be utilized in genome studies using the WGS strategy.

Several investigations have determined a genome size range of 294–782 Mb for at least 33 rose species and several cultivars [55]. This observed variability in genome size is not likely due to differences in gene numbers but rather to variations in non-coding sequences such as the intron size [56], and a variety of other factors, including the copy number of TEs, the amount or size of SSRs, the size of inter-enhancer spacers, and the number of pseudogenes [57]. For example, the observed genome size difference between apple and pear is mainly due to repetitive sequences that are predominantly contributed by TEs, whereas the size of the genic regions is similar in both species [18]. In addition, different TE compositions, especially the composition of LTRs, resulting from TE multiplication, may cause genome size changes, which might have large effects on speciation [58, 59]. In the present study, The ratio (0.71 to 1) of *gypsy* to *copia* LTRs in *R*. *roxburghii* was remarkably lower than that observed in peach (1.16 to 1) [17], strawberry (1.20 to 1) [15], pear (1.99 to 1) [19], and apple (4.58 to 1) [14]. These results could contribute to the understanding of Rosaceae genome evolution [60].

The TE content in *R*. *roxburghii* was 29.20%, which was similar to that of peach (29.60%) [17]. In addition, the amount of LTRs, which comprised 16.74% of the *R*. *roxburghii* genome, was similar to that of strawberry (~16%) [15]. However, the genome size of *R*. *roxburghii* was ~2-fold larger than that of the two species. This difference in genome size might not be due to the amount of TEs or LTRs, but the composition of LTRs. Meanwhile, SSRs comprised 1.15% of the *R*. *roxburghii* genome, which was significantly larger than that observed in apple (0.27) [14], *Pyrus bretschneideri* (0.22) [18], and *Pyrus communis* (0.04) [19], and might have potentially led to the genome expansion of *R*. *roxburghii*.

Genomic SSR markers, reliable, highly polymorphic, often multi-allelic, and easy to amplify, are widely used in genetic diversity, genetic map construction and so on [53]. However, the lack of available genomic resources in *R*. *roxburghii* impeded the use of microsatellite markers. To date, the limited EST-SSR markers were developed for *R*. *roxburghii* [9], but no genome-wide SSR markers have been published. Presently, the genome survey based on NGS is an especially useful method to explore SSR markers for tree crops [61].

Compared to fruits [9, 34], *R*. *roxburghii* leaves undergo a higher level of active oxidation loss and recycling of AsA. The L-galactose pathway is considered as the dominant route for AsA biosynthesis in several plant species [62], and *GGP* may play a key role in the L-galactose pathway in *R*. *roxburghii* fruits [9]. In the present study, *GGP* was not highly expressed in AsA-abundant aged leaves, although the level of *GLDH* expression was similar to the variable pattern of T-AsA content. Besides, the discovery of *GUR* and *MIOX* genes in the present study suggests that *R*. *roxburghii* can use GalUA or *myo-*inositol as an initial substrate in AsA biosynthesis, implying that multiple pathways were involved in AsA metabolism in *R*. *roxburghii* leaves. In addition, *GUR* via the GalUA pathway played important roles in AsA biosynthesis in strawberry fruits [63]. *APX*, which encodes a well-recognized enzyme, catalyzes the oxidation of AsA with high specificity, and *AAO*, which encodes another vital redox enzyme, also catalyzes the oxidation of apoplast AsA in the presence of oxygen. These two upregulated genes might have caused DHA accumulation in mature leaves (Fig 8).

This is the first report of genome-wide characterization in the genus *Rosa*. Among the 100–250 species in this genus, *R*. *roxburghii* is most important in terms of its horticultural, nutritional, and medicinal value. However, its limited genomic information has constrained genetic studies of *R*. *roxburghii*. A total of 167,859 SSRs and 22,721 genes derived from the *R*. *roxburghii* genome survey could help in the construction of high-density linkage maps and in conducting gene-based association studies. In addition, the generated dataset could contribute to the understanding of Rosaceae genome evolution. Evaluation of the expression of candidate genes involved in AsA metabolism may improve our understanding of the molecular mechanisms underlying ascorbate accumulation in *R*. *roxburghii*.

## Supporting Information

S1 Fig Gene assisgnment to KOG functional categories in *R*. *roxburghii*. (TIF)

S2 Fig Evolutionary relationships among species. (TIF)

S1 Table Sequences of specific primers used for quantitative real-time PCR. (XLS)

S2 Table Comparison of SOAPdenovo and Abyss for assembly. (XLS)

S3 Table Occurrence of SSR motifs in Genome Survey to *R*. *roxburghii*. (XLS)

S4 Table Number of genes mapped onto KEGG pathways. (XLS)

S5 Table Genes involved in the ascorbate metabolism. (XLS)

## References

[1] Smulders MJM, Arens P, Koning-Boucoiran CFS, Gitonga VW, Krens FA, Tanassov AA, et al Rosa In: Kole C, editor. Wild crop relatives: genomic and breeding resources, plantation and ornamental crops. Berlin: Springer; 2012 pp. 277–296.

[2] Darlington CD, Wylie AP. Chromosome atlas of flowering plants 2nd ed. London: Allen & Unwin; 1955.

[3] He ZF, Niu AZ, Xiang XH, Wang SM. A study on the nutrition and variation in the vitamin C content in the fruits of *Rosa roxburghii* Tratt. Acta Horticulturae Sinica. 1984; 11: 271–273.

[4] Liu QL, Fan WG, AN HM. Study on the extraction of water-soluble polysaccharides and total flavone from *Rosa roxburghii* leaves. Journal of Mountain Agriculture and Biology. 2005; 24: 522–526.

[5] An HM, Chen LG, Fan WG, Liu Qinglin. Ascorbate contents and activities of some antioxidant enzymes during senescence of *Rosa roxburghii* leaves. Acta Horticulturae Sinica. 2005; 32: 994–997.

[6] Zhu WF, Xiang XH, Yang SX, Mo QQ, Gao XF. Investigation of chestnut rose germplasms, growth and development, and vitamin C content in deferent ecosystems of Guizhou Province (China). Journal of Guizhou Agricultural College. 1984; 3: 1–13.

[7] Fan WG, Xiang XH, An HM, Liu JP. A new *Rosa roxburghii* cultivar ‘Guinong 5’. Acta Horticulturae Sinica. 2011; 38: 1609–1610.

[8] Wen XP, Pang XM, Deng XX. Characterization of genetic relationships of *Rosa roxburghii* Tratt and its relatives using morphological traits, RAPD and AFLP markers. J Hortic Sci Biotech. 2004; 79: 189–196.

[9] Yan XQ, Zhang X, Lu M, He Y, An HM. *De novo* sequencing analysis of the *Rosa roxburghii* fruit transcriptome reveals putative ascorbate biosynthetic genes and EST-SSR markers. Gene. 2015; 561: 54–62. 10.1016/j.gene.2015.02.054 25701597

[10] Davey MW, Montagr MV, Inzé D, Sanmartin M, Kanellis A, Smirnoff N et al Plant L-ascorbic acid: chemistry, function, metabolism, bioavailability and effects of processing. J Sci Food Agr. 2000; 80: 825–860.

[11] Conklin PL, Barth C. Ascorbic acid, a familiar small molecule intertwined in the response of plants to ozone, pathogens, and the onset of senescence. Plant Cell Environ. 2004; 27: 959–970.

[12] Wheeler GL, Jones MA, Smirnoff N. The biosynthetic pathway of vitamin C in higher plants. Nature. 1998; 393: 365–369. 9620799 10.1038/30728

[13] Chen Z, Young TE, Ling J, Chang SC, Gallie DR. Increasing vitamin C content of plants through enhanced ascorbate recycling. Proc Natl Acad Sci USA. 2003; 100: 3525–3530. 12624189 10.1073/pnas.0635176100 PMC152326

[14] Velasco R, Zharkikh A, Affourtit J, Dhingra A, Cestaro A, Kalyanaraman A, et al The genome of the domesticated apple (*Malus × domestica* Borkh.). Nat Genet. 2010; 42: 833–841. 10.1038/ng.654 20802477

[15] Shulaev V, Sargent DJ, Crowhurst RN, Mockler TC, Folkerts O, Delcher AL, et al The genome of woodland strawberry (*Fragaria vesca*). Nat Genet. 2011; 43: 109–116. 10.1038/ng.740 21186353 PMC3326587

[16] Zhang Q, Chen W, Sun L, Zhao F, Huang B, Yang W et al The genome of *Prunus mume*. Nat Commun. 2012; 3: 187–190. 10.1038/ncomms2290 PMC3535359 23271652

[17] The International Peach Genome Initiative. The high-quality draft genome of peach (*Prunus persica*) identifies unique patterns of genetic diversity, domestication and genome evolution. Nat Genet. 2013; 45: 487–494. 10.1038/ng.2586 23525075

[18] Wu J, Wang Z, Shi Z, Zhang S, Ming R, Zhu S, et al The genome of the pear (*Pyrus bretschneideri* Rehd.). Genome Res. 2013; 23: 396–408. 10.1101/gr.144311.112 23149293 PMC3561880

[19] Chagné D, Crowhurst RN, Pindo M, Thrimawithana A, Deng C, Ireland H, et al The draft genome sequence of European pear (*Pyrus communis* L. 'Bartlett'). PLoS One. 2014; 9: e92644 10.1371/journal.pone.0092644 24699266 PMC3974708

[20] Varshney RK, Chen W, Li Y, Bharti AK, Saxena RK, Schlueter JA. Draft genome sequence of pigeonpea (*Cajanus cajan*), an orphan legume crop of resource-poor farmers. Nat Biotechnol. 2012; 30: 83–89. 10.1038/nbt.2022 22057054

[21] Li Y, Hu Y, Bolund L, Wang J. State of the art *de novo* assembly of human genomes from massively parallel sequencing data. Hum Genet. 2010; 4: 271–277. 10.1186/1479-7364-4-4-271 PMC3525208 20511140

[22] Xu Z, Wang H. LTR_FINDER: an efficient tool for the prediction of full-length LTR retrotransposons. Nucleic Acids Res. 2007; 35:W265–W268. 17485477 10.1093/nar/gkm286 PMC1933203

[23] Han Y, Wessler SR. MITE-Hunter: a program for discovering miniature inverted-repeat transposable elements from genomic sequences. Nucleic Acids Res. 2010; 38: e199 10.1093/nar/gkq862 20880995 PMC3001096

[24] Price AL, Jones NC, Pevzner PA. De novo identification of repeat families in large genomes. Bioinformatics. 2005; 21: i351–i358. 15961478 10.1093/bioinformatics/bti1018

[25] Edgar RC, Myers EW. PILER: identification and classification of genomic repeats. Bioinformatics. 2005; 21: i152–i158. 15961452 10.1093/bioinformatics/bti1003

[26] Wicker T, Sabot F, Hua-Van A, Bennetzen JL, Capy P, Chalhoub B et al A unified classification system for eukaryotic transposable elements. Nat Rev Genet. 2007; 8: 973–982. 17984973 10.1038/nrg2165

[27] Jurka J, Kapitonov VV, Pavlicek A, Klonowski P, Kohany O, Walichiewicz J. Repbase Update, a database of eukaryotic repetitive elements. Cytogenet Genome Res. 2005; 110: 462–467. 16093699 10.1159/000084979

[28] Chen N. Using RepeatMasker to identify repetitive elements in genomic sequences. Current Protocols in Bioinformatics. 2004; 5: 4. 10. 11–14. 10. 14. 10.1002/0471250953.bi0410s05 18428725

[29] Kofler R, Schlotterer C, Lelley T. SciRoKo: a new tool for whole genome microsatellite search and investigation. Bioinformatics. 2007; 23: 1683–1685. 17463017 10.1093/bioinformatics/btm157

[30] Salamov AA, Solovyev VV. *Ab initio* gene finding in *Drosophila* genomic DNA. Genome Res. 2000; 10: 516–522. 10779491 10.1101/gr.10.4.516 PMC310882

[31] Hirakawa H, Okada Y, Tabuchi H, Shirasawa K, Watanabe A, Tsuruoka H et al Survey of genome sequences in a wild sweet potato, Ipomoea trifida (H. B. K.) G. Don. DNA Res. 2015; 22: 171–179. 10.1093/dnares/dsv002 25805887 PMC4401327

[32] Li L, Stoeckert CJ, Roos DS. OrthoMCL: identification of ortholog groups for eukaryotic genomes. Genome Res. 2003; 13: 2178–2189. 12952885 10.1101/gr.1224503 PMC403725

[33] Guindon S, Dufayard JF, Lefort V, Anisimova M, Hordijk W, Gascuel O. New algorithms and methods to estimate maximum-likelihood phylogenies: assessing the performance of PHYML 3.0. Syst Biol. 2010; 59: 307–321. 10.1093/sysbio/syq010 20525638

[34] An HM, Fan WG, Chen LG, Asghar S, Liu GQ. Molecular characterisation and expression of L-galactono-1, 4-lactone dehydrogenase and L-ascorbic acid accumulation during fruit development in *Rosa roxburghii*. J Hortic Sci Biotech. 2007; 82:627–635.

[35] Nygaard S, Zhang G.J, Schiøtt M, Li C, Wurm Y, Hu H et al The genome of the leaf-cutting ant *Acromyrmex echinatior* suggests key adaptations to advanced social life and fungus farming. Genome Res. 2011; 21: 1339–1348. 10.1101/gr.121392.111 21719571 PMC3149500

[36] Smith CR, Smith CD, Robertson HM, Helmkampf M, Zimin A, Yandell M, et al Draft genome of the red harvester ant *Pogonomyrmex barbatus*. Proc Natl Acad Sci USA. 2011; 108: 5667–5672. 10.1073/pnas.1007901108 21282651 PMC3078412

[37] The Potato Genome Sequencing Consortium. Genome sequence and analysis of the tuber crop potato. Nature. 2011; 475:1–9. 10.1038/nature10158 21743474

[38] Werren JH, Richards S, Desjardins CA, Niehuis O, Gadau J, Colbourne JK, et al Functional and evolutionary insights from the genomes of three parasitoid *nasonia* species. Science. 2010; 327: 343–348. 10.1126/science.1178028 20075255 PMC2849982

[39] Al-Dous EK, George B, Al-Mahmoud ME, Al-Jaber MY, Wang H, Salameh YM, et al *De novo* genome sequencing and comparative genomics of date palm (*Phoenix dactylifera*). Nat Biotechnol. 2011; 29: 521–527. 10.1038/nbt.1860 21623354

[40] Renfree MB, Papenfuss AT, Deakin JE, Lindsay J, Heider T, Belov K et al Genome sequence of an Australian kangaroo, *Macropus eugenii*, provides insight into the evolution of mammalian reproduction and development. Genome Biol. 2011; 12: R81 10.1186/gb-2011-12-8-r81 21854559 PMC3277949

[41] Aird D, Ross MG, Chen WS, Danielsson M, Fennell T, Russ C, et al Analyzing and minimizing PCR amplification bias in Illumina sequencing libraries. Genome Biol. 2011; 12: R18 10.1186/gb-2011-12-2-r18 21338519 PMC3188800

[42] Sato S, Nakamura Y, Kaneko T, Asamizu E, Kato T, Nakao M, et al Genome structure of the legume, *Lotus japonicas*. DNA Res. 2008; 15: 227–239. 10.1093/dnares/dsn008 18511435 PMC2575887

[43] The Tomato Genome Consortium. The tomato genome sequence provides insights into fleshy fruit evolution. Nature. 2012; 485: 635–641. 10.1038/nature11119 22660326 PMC3378239

[44] Sonah H, Deshmukh RK, Sharma A, Sing VP, Gupta DK, Gacche RN, et al Genome-wide distribution and organization of microsatellites in plants: an insight into marker development in *Brachypodium*. PLoS One. 2011; 6:e21298 10.1371/journal.pone.0021298 21713003 PMC3119692

[45] Zhou W, Hu YY, Sui ZH, Fu F, Wang JG, Chang LP, et al Genome survey sequencing and genetic background characterization of *Gracilariopsis lemaneiformis* (Rhodophyta) based on next-generation sequencing. PLoS One. 2013; 8: e69909 10.1371/journal.pone.0069909 23875008 PMC3713064

[46] Arumuganathan K, Earle ED. Nuclear DNA content of some important plant species. Plant Mol Biol Rep. 1991; 9:208–218.

[47] Huang SW, Li RQ, Zhang ZH, Li L, Gu XF, Fan W, et al The genome of the cucumber, *Cucumis sativus* L. Nat Genet. 2009; 41:1275–1281. 10.1038/ng.475 19881527

[48] Jiao Y, Jia HM, Li XW, Chai ML, Jia HJ, Chen Z, et al Development of simple sequence repeat (SSR) markers from a genome survey of Chinese bayberry (*Myrica rubra*). BMC Genomics. 2012; 13: 201 10.1186/1471-2164-13-201 22621340 PMC3505174

[49] Yokoya K, Roberts AV, Mottley J, Lewis R, Brandham PE. Nuclear DNA amounts in roses. Ann Bot. 2000; 85: 557–561.

[50] Dolezel J, Bartos J, Voglmayr H, Greilhuber J. Nuclear DNA content and genome size of trout and human. Cytometry. 2003; 51:127–128. 12541287 10.1002/cyto.a.10013

[51] Xu Q, Chen LL, Ruan X, Chen D, Zhu A, Chen C, et al The draft genome of sweet orange (*Citrus sinensis*). Nat Genet. 2012; 45: 59–66. 10.1038/ng.2472 23179022

[52] Bryc K, Patterson N, Reich D. A novel approach to estimating heterozygosity from low-coverage genome sequence. Genetics. 2013; 195: 553–561. 10.1534/genetics.113.154500 23934885 PMC3781980

[53] Varshney RK, Graner A, Sorrells ME. Genic microsatellites markers in plants: features and application. Trends Biotechnol. 2005; 23: 48–55. 15629858 10.1016/j.tibtech.2004.11.005

[54] Tuskan GA, Difazio S, Jansson S, Bohlmann J, Grigoriev I, Hellsten U, et al The genome of black cottonwood, *Populus trichocarpa* (Torr. & Gray). Science. 2006; 313: 1596–1604. 16973872 10.1126/science.1128691

[55] Debener T, Linde M. Exploring complex ornamental genomes: the rose as a model plant. Crit Rev Plant Sci. 2009; 28: 267–280.

[56] Vinogradov AE. Intron-genome size relationship on a large evolutionary scale. J Mol Evol. 1999; 49:376–384. 10473779 10.1007/pl00006561

[57] Petrov DA. Evolution of genome size: new approaches to an old problem. Trends Genet. 2001; 17: 23–28. 11163918 10.1016/s0168-9525(00)02157-0

[58] Bennetzen JL. Comparative sequence analysis of plant nuclear genomes: microcolinearity and its many exceptions. Plant Cell. 2000; 12: 1021–1029. 10899971 10.1105/tpc.12.7.1021 PMC149046

[59] Zhang GY, Liu X, Quan ZW, Cheng SF, Xu X, Pan SK, et al Genome sequence of foxtail millet (*Setaria italica*) provides insights into grass evolution and biofuel potential[J]. Nature biotechnology, 2012, 30(6): 549–554. 10.1038/nbt.2195 22580950

[60] Kraaijeveld K. Genome size and species diversification. Evol Biol. 2010; 37: 227–233. 22140283 10.1007/s11692-010-9093-4 PMC3227167

[61] Ravishankar KV, Dinesh MR, Nischita P, Sandya BS. Development and characterization of microsatellite markers in mango (*Mangifera indica*) using next-generation sequencing technology and their transferability across species. Mol Breeding. 2015; 35: 93.

[62] Linster CL, Gomez TA, Christensen KC, Adler LN, Young BD, Brenner C et al *Arabidopsis VTC2* encodes a GDP-L-galactose phosphorylase, the last unknown enzyme in the Smirnoff-Wheeler pathway to ascorbic acid in plants. J Biol Chem. 2007; 282: 18879–18885. 17462988 10.1074/jbc.M702094200 PMC2556065

[63] Cruz-Rus E, Amaya I, Sánchez-Sevilla JF, Botella MA, Valpuesta V. Regulation of L-ascorbic acid content in strawberry fruits. J Exp Bot. 2011; 62: 4191–4201. 10.1093/jxb/err122 21561953 PMC3153677

