# Supplementary figures and images for: Genome Survey Sequencing for the Characterization of the Genetic Background of Rosa roxburghii Tratt and Leaf Ascorbate Metabolism Genes
Source: PLoS One. 2016 Feb 5;11(2):e0147530. doi: 10.1371/journal.pone.0147530 (PMC4743950; doi:10.1371/journal.pone.0147530)

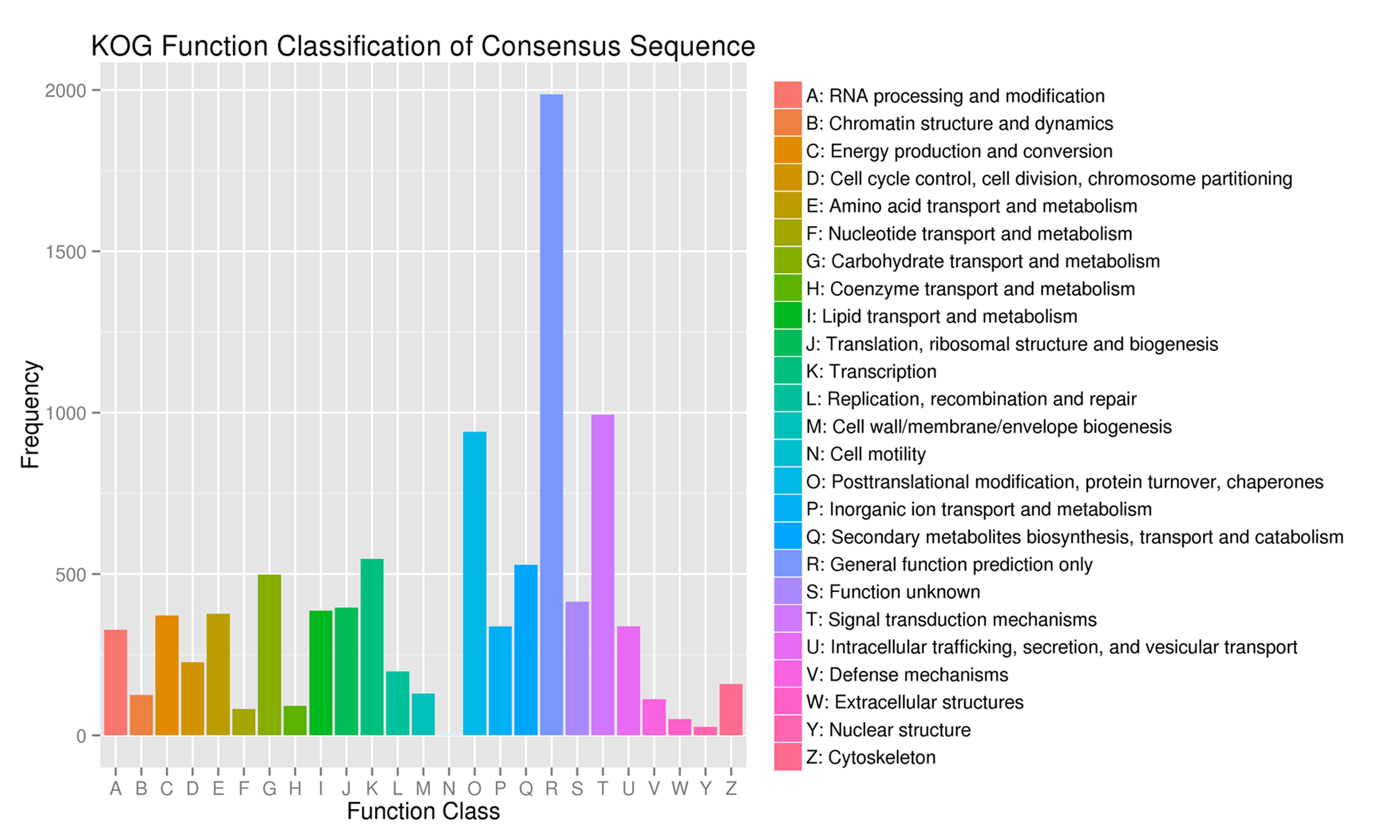

Supplement: S1 Fig — (TIF) [file pone.0147530.s001.tif]

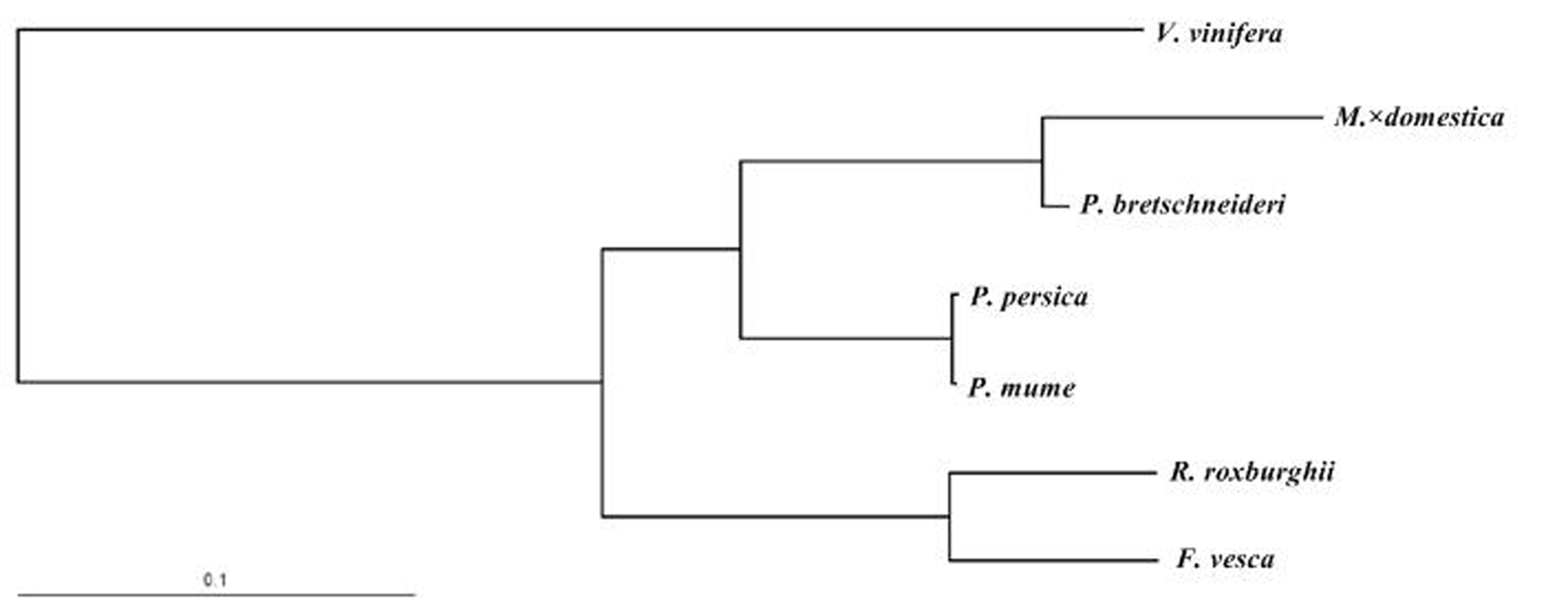

Supplement: S2 Fig — (TIF) [file pone.0147530.s002.tif]
